# Tick-borne Pathogen Detection and Its Association with Alterations in Packed Cell Volume of Dairy Cattle in Thailand

**DOI:** 10.3390/ani13182844

**Published:** 2023-09-07

**Authors:** Paul Franck Adjou Moumouni, Eloiza May Galon, Maria Agnes Tumwebaze, Benedicto Byamukama, Ruttayaporn Ngasaman, Saruda Tiwananthagorn, Ketsarin Kamyingkird, Tawin Inpankaew, Xuenan Xuan

**Affiliations:** 1 National Research Center for Protozoan Diseases, Obihiro University of Agriculture and Veterinary Medicine, Obihiro 080-8555, Japan; chakirou82@yahoo.fr (P.F.A.M.); tumwebazeaggie@gmail.com (M.A.T.); benards.benedicto4@gmail.com (B.B.); 2 College of Veterinary Medicine and Biomedical Sciences, Cavite State University, Indang 4122, Philippines; 3 Faculty of Veterinary Science, Prince of Songkla University, Hat Yai 90110, Thailand; ruttayaporn.n@gmail.com; 4 Department of Veterinary Biosciences and Public Health, Faculty of Veterinary Medicine, Chiang Mai University, Chiang Mai 50100, Thailand; saruda.t@cmu.ac.th; 5 Department of Parasitology, Faculty of Veterinary Medicine, Kasetsart University, Lad Yao, Chatuchak, Bangkok 10900, Thailand; ketsarinkamy@hotmail.com

**Keywords:** *Anaplasma*, *Babesia*, *Theileria*, packed cell volume, dairy cattle, Thailand

## Abstract

**Simple Summary:**

Tick-borne diseases adversely impact bovine health, leading to huge financial losses for farmers. Timely and accurate disease diagnosis is key in managing bovine herds, particularly in production animals such as dairy cattle. In the present study, we molecularly detected tick-borne pathogens (TBPs) in dairy cattle from selected provinces in the northern and western parts of Thailand. Specifically, common TBP infection was widespread in the sampled animals. Two detection tools were also compared for pathogen detection. Furthermore, cattle positive for tick-borne infections had notably lower hematocrit values. The results of this study emphasize the importance of regular tick-borne surveillance and its possible clinical impact on economically important dairy cattle in Thailand.

**Abstract:**

Tick-borne diseases (TBDs) massively impact bovine production. In endemic countries, animals are often subclinically infected, showing no signs of the illness. Anemia is a hallmark of TBDs, but there is inadequate information on its presence in infected Thai cattle. In the present study, 265 cattle from four provinces in Thailand were surveyed to identify tick-borne pathogens (TBPs) and to evaluate the changes in the packed cell volume (PCV) values associated with detection. Microscopy and polymerase chain reaction (PCR) were also compared for TBP detection. *Babesia*/*Theileria*/*Hepatozoon* was detected in 33.58% (89/265) of the cattle samples. Specifically, *Babesia bovis* (9/265), *B. bigemina* (12/265), *Theileria orientalis* (62/265), and *Anaplasma marginale* (50/265) were identified using species-specific assays. Significant decreases in the mean PCV levels were observed in cattle that were positive for at least one TBP (*p* < 0.001), *Babesia*/*Theileria*/*Hepatozoon* (*p* < 0.001), *T. orientalis* (*p* < 0.001), and *A. marginale* (*p* = 0.049). The results of PCR and microscopy for the detection of TBPs suggested slight and fair agreement between the two detection tools. The present findings contribute to a better understanding of TBDs in the field and shall facilitate the formulation of effective control for TBDs in Thailand.

## 1. Introduction

Dairy farming is an essential aspect of livestock raising, providing a stable economic enterprise scheme for smallholder farmers and a source of nutritious dairy products. In Southeast Asian countries such as Thailand, the dairy industry is largely composed of dairy farms that operate on a small scale [1]. The growth potential of dairy farming is well recognized; thus, initiatives to improve productivity, such as breed improvement programs, have been carried out. However, alongside the advancement of the dairy sector, constraints have continuously hampered its development. One of the glaring problems encountered by dairy farms using genetically improved stocks for dairy farming is the relatively greater susceptibility to infectious diseases compared to native stocks, specifically diseases that are carried by ticks and other vectors. Tick-borne diseases (TBDs) have been a major threat in the global cattle-raising industry, forcing farmers to shoulder US$20–30 billion in losses annually [2,3].

The most common bovine TBDs in Thailand are theileriosis, babesiosis, and anaplasmosis. Theileriosis is among the most serious TBDs. Economically notable species affecting bovines include the *Theileria orientalis* group, *T. annulata*, and *T. parva*, the known agents of oriental theileriosis, tropical theileriosis, and East Coast Fever, respectively [4]. Of these, *T. orientalis* has been confirmed to be present in Thailand [5,6,7]. *T. orientalis* infection in cattle may cause pyrexia, anemia, icterus, weakness, pallor, lethargy, abortion, and in severe outbreaks death [4]. Babesiosis is a major TBD affecting bovines, especially those in tropical and subtropical areas of the world. *Babesia bovis* and *B. bigemina* are the two most important species, as they account for the majority of bovine babesiosis clinical cases. Other species infecting cattle include *B. divergens* and *B. ovata*, which are prevalent in Europe and East Asia [8], respectively, and the recently discovered *B. naoakii* [9]. In endemically stable countries, clinical cases of bovine babesiosis are rare and usually display as fever, anemia, and hemoglobinuria, and in the case of *B. bovis*, nervous and respiratory signs lead to fatalities. In Thailand, several *Babesia* species have been detected in cattle using molecular techniques [6,10,11,12,13]. Anaplasmosis is a disease resulting from *Anaplasma* spp. infection, including *A. marginale*. This pathogen is transmitted not only by ticks but also through mechanical (e.g., biting flies, lice, and contaminated fomites) and transplacental means [14]. Infected cattle show signs of inappetence, weakness, anorexia, pyrexia, anemia, paleness, icterus, and reproductive problems such as abortion and infertility [14,15]. After initial infection, cattle are persistently infected for life and act as carriers of the pathogen [16].

The abovementioned parasites invade the host red blood cells (RBCs), and their proliferation involves the destruction of RBCs. Subsequently, a cardinal sign of these TBDs is anemia, which is usually seen in severe clinical cases of theileriosis, babesiosis, and anaplasmosis. The impact of anemia on animals such as cattle is substantial, as it can reduce meat and milk production [17]. and in severe cases, it can lead to mortalities within herds. Although the risk of clinical cases of TBDs is low in endemic areas [18], the possible emergence of pathogenic isolates of TBPs can devastate dairy herds in Thailand.

Prompt and accurate identification of agents is vital in the control of TBDs in the field. Traditional diagnostic tools (microscopy of blood smears, inoculation, in vitro culture) and immunological methods (status of exposure through the detection of specific antibodies) are usually lengthy and laborious. Moreover, these tools have a variety of sensitivity (particularly in low-parasitemia/bacteremia animals) and specificity issues (differential identification of agents), often making the diagnosis imprecise [19]. The use of the nucleic-acid-based platform, such as isothermal and polymerase chain reaction (PCR) assays, has addressed these issues due to its improved analytical and diagnostic sensitivity and specificity.

This study aimed to identify TBPs infecting dairy cattle in selected provinces in northern and western Thailand and evaluate the association of TBP detection with the packed cell volume (PCV) of animals. In addition, the agreement between two tools in detecting TBPs, namely, microscopy and PCR, was compared.

## 2. Materials and Methods

### 2.1. Ethical Statements

The farm owners were briefed and informed on the purpose of the study prior to the collection of samples. The animals were handled and restrained with utmost care to minimize discomfort. Procedures employed in the survey adhered to the animal welfare laws in Thailand and the ethical guidelines for the use of animal samples implemented by Obihiro University of Agriculture and Veterinary Medicine (animal experiment approval 22-23; DNA experiment approval 20-07, 20-08; approval date: 1 April 2023)

### 2.2. Background of Farms and Sampled Cattle

A total of 20 farms located in 4 provinces, namely, Lampang (N = 10), Lamphun (N = 8), Nakhon Pathom (N = 1), and Kanchanaburi (N = 1), were surveyed in the present study (Table 1). The herd sizes in the surveyed farms ranged from 4 to 93 head of Holstein Friesian or crossbred (Holstein Friesian and native) cattle. Eleven farms were medium-scale dairy farms (20 to 50 head), while 5 and 4 farms were large-scale (>50 head) and small-scale farms (<20 head), respectively (Table 1). Data on the management practices relating to parasite and tick controls were only available for 18 farms from Lampang and Lamphun. Lampang farmers free-grazed their livestock, whereas Lamphun farmers practiced stall feeding. Of the 10 farms in Lampang, all farmers were aware of the impact of TBDs, and 70% of them implemented tick control measures. On the other hand, only 3 of the Lamphun farms were aware of TBDs, and only half of them practiced tick countermeasures (Table 1).

### 2.3. Study Site and Blood Sample Collection

**Figure 1:**
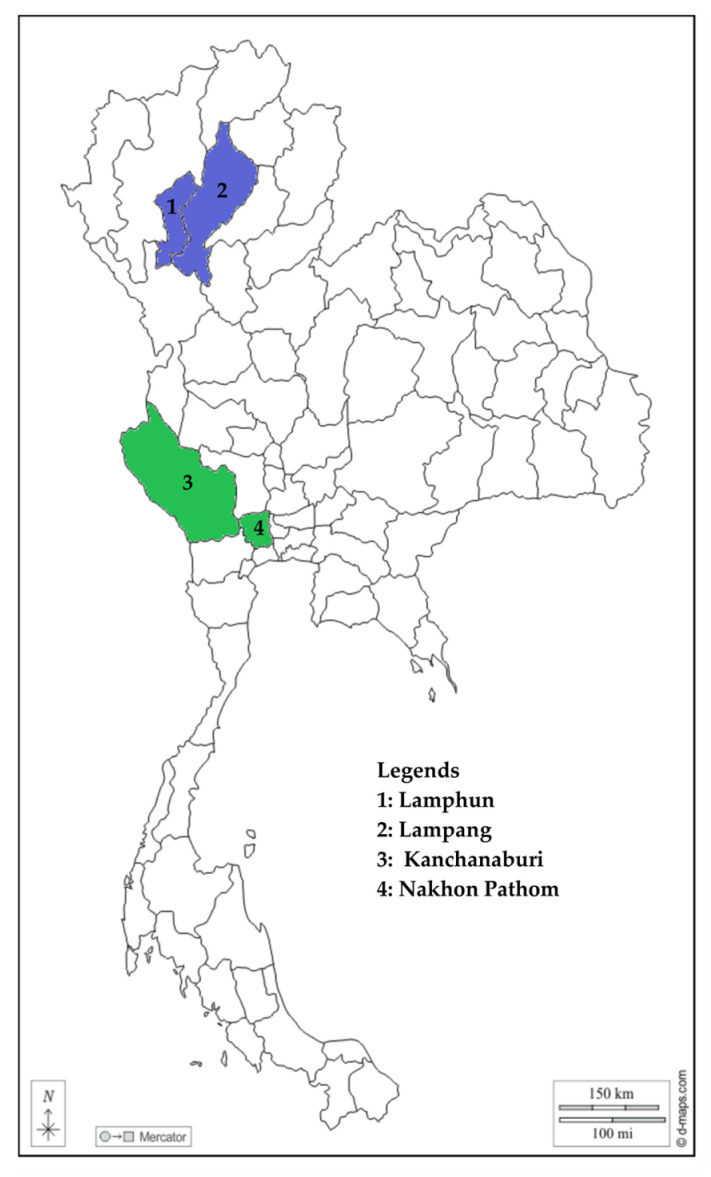
Map of Thailand with provinces (shaded) where the surveyed farms are located. Purple-shaded provinces are from the northern part, while green-shaded provinces are from the western part. The map was adopted and modified from d-maps.com (accessed on 3 September 2023).

From September to December 2017, a total of 265 Holstein cattle from 20 dairy farms were the subjects of the current study. Farms were selected based on their willingness to take part in the study and as suggested by local collaborators. Animals, however, were randomly selected. These cattle were all clinically healthy with no obvious signs of disease. Approximately 10% of the animal population of the farm was used as the basis for the sample number per sampling site. The samples were from the provinces of Lampang (n = 84), Lamphun (n = 51), Nakhon Pathom (n = 70), and Kanchanaburi (n = 60) (Figure 1). Whole blood samples (5 mL) were aseptically collected in ethylenediaminetetraacetic acid-anticoagulated vacutainers. The samples were kept at 4 °C until further processing.

### 2.4. Hematologic Analysis and Microscopic Examination

The PCV values of the samples were analyzed using an automatic hematologic analyzer. Thin blood smears were prepared in glass slides, air-dried, fixed with methanol, and stained with 20% Giemsa in 1 × phosphate-buffered saline solution for 30 to 45 min. Slides were observed using a light microscope under an oil-immersed 100× magnification lens (Nikon Eclipse, Tokyo, Japan). Common bovine blood parasites (*Babesia*, *Theileria*, and *Anaplasma*) were searched for in at least 1000 erythrocytes.

### 2.5. Blood Sample Processing and PCR Tests of DNA Samples

Approximately 200 µL of whole blood was used for isolating the genomic DNA (gDNA) using the column-based QIAamp^®^ Mini Blood Kit following the manufacturer’s instructions (Qiagen, Hilden, Germany). The samples were eluted to a final volume of 100 µL. The extracted DNA samples were stored at −30 °C until the molecular screening.

A PCR test was performed to molecularly identify samples positive for BTH (*Babesia, Theileria,* and *Hepatozoon*) 18S rRNA gene [20]. Then, subsequent piroplasma identification was also conducted using species-specific assays for *B. bigemina* [21], *B. bovis* [22], and *T. orientalis* [23], with gene targets, namely, rhoptry-associated protein-1a (RAP-1a), spherical body protein-2 (SBP-2), and major piroplasm surface protein (MPSP), respectively. In addition, *A. marginale* was detected using a species-specific, nested assay targeting the heat-shock chaperon gene (*groEL)* [24]. Details on the primer sets and assays used are listed in Table S1. The PCR was run at a final volume setup of 10 µL, comprising 1 × ThermoPol^®^ reaction buffer (New England Biolabs, Ipswich, MA, USA), 2 μM each of dNTP in a solution mix, 0.25 U *Taq* DNA polymerase (New England Biolabs), 1.5 μL gDNA sample, 2 μM of each primer, and 6.85 μL double-distilled water. For the nested assays, 1.5 μL of PCR product obtained from the first round of amplification was used for the second-round reaction. The thermocycling conditions from the referenced studies were followed, except for the annealing and extension temperatures and duration, which were modified based on the provided conditions in the *Taq* polymerase kit (New England Biolabs). Controls were run alongside the samples to ensure the absence of contamination. gDNA of *Babesia* (*B. bigemina* Argentina strain and *B. bovis* Texas strain), and bovine DNA samples that were confirmed positive for *T. orientalis* and *A. marginale* by sequencing were used as positive controls in the assays, while the negative control was double-distilled water. PCR products were gel-electrophoresed on a 1.5% agarose in TAE buffer solution, stained with ethidium bromide (Nacalai Tesque, Kyoto, Japan), then viewed under a UV transilluminator (Atto, Tokyo, Japan). Positive samples were confirmed with the presence of a band similar to that of the corresponding positive control samples.

### 2.6. Statistical Analyses

The various analyses were performed using GraphPad Prism 8.0.2 (GraphPad Software, San Diego, CA, USA) and Microsoft Excel. Pearson’s chi-squared test (χ2) was run to determine the association between PCR positivity and sample location. The datasets for mean PCV values were tested for normality prior to running an unpaired Student’s *t*-test or a Mann–Whitney U test to evaluate the changes between positive and negative samples. The significance threshold for *p* values was set at <0.05. The degree of agreement between microscopy and PCR results was determined based on Cohen’s kappa [25]. The kappa values were interpreted as: no agreement (<0), slight agreement (0–0.20), fair agreement (0.21–0.40), moderate agreement (0.41–0.60), substantial agreement (0.61–0.80), and almost perfect agreement (0.81–1.00) [25].

## 3. Results

### 3.1. Tick-borne Pathogen Detection by PCR

Generic PCR screening showed that 33.58% (89/265) were positive for BTH, with the highest detection rate observed in Lampang (63.10%; 53/84) and Nakhon Pathom (32.86%; 23/70) (Table 2). Species-specific assays revealed that *B. bigemina, B. bovis, T. orientalis*, and *A. marginale* were detected in 4.53%, 3.40%, 23.40%, and 18.87% of cattle samples, respectively. Compared with other provinces, samples from Lampang had significantly higher detection rates for all the detected pathogens (Table 2). *B. bigemina* and *B. bovis* were detected in three provinces, while *T. orientalis* and *A. marginale* were detected in all surveyed provinces. In addition, all screened pathogens were present in cattle samples from Lampang and Nakhon Pathom provinces. Based on the results of the species-specific PCR assays, 74 samples were positive for only 1 TBP, while 26 were positive for 2 or more TBPs: 20, 5, and 1 sample/s had dual, triple, and quadruple infections, respectively (Table 3). Specifically, coinfection with *T. orientalis* and *A. marginale* (n = 13) was recorded as the most common combination of TBPs in the coinfected samples.

### 3.2. Agreement between PCR and Microscopic Examination Results

Table 4 shows the results obtained by comparing PCR and microscopy methods for TBP detection. The microscopic examination searched for *Babesia*, *Theileria*, and *Anaplasma* in 176 samples only. Therefore, we excluded the samples without microscopy data for analysis. For piroplasma, the PCR targeted the BTH 18S rRNA gene (*Babesia/Theileria/Hepatozoon*), while the microscopy data were the pooled results for *Babesia* and *Theileria*. Based on Cohen’s kappa index, piroplasma (κ = 0.20) and *Babesia* (κ = 0.11) showed slight agreement between PCR and microscopy results, while *Theileira* and *Anaplasma* both recorded a κ value of 0.21, indicating a fair agreement between the two detection tools (Table 4).

### 3.3. PCV Alterations Associated with Tick-Borne Pathogen Positivity

As anemia is one of the hallmarks of TBDs, we analyzed if PCR-positive samples had altered PCV values compared with the PCR-negative samples (Table 5). We excluded in the analysis the PCR results with fewer than 15 values per group (i.e., data on *B. bigemina* and *B. bovis*) and one clotted sample that had an incorrect reading to avoid biases. The mean PCV of samples positive for at least one TBP (27.59 ± 5.21) was significantly lower than the mean PCV of uninfected samples (30.54 ± 4.52). Similar trends were observed in the mean PCV of BTH 18S rRNA-positive (27.18 ± 5.39) and *T. orientalis*-positive (26.90 ± 5.44) samples, which were significantly decreased compared with those of PCR-negative samples (30.11 ± 4.62 and 29.82 ± 4.76, respectively). The mean PCV of *A. marginale*-positive (27.84 ± 4.92) samples was notably lower than that of the uninfected samples (29.43 ± 5.07).

## 4. Discussion

The adverse impact of TBDs on bovine health, mainly on dairy herds, has led to not only financial losses but more importantly to opportunity costs that have hampered the improvement of the growing dairy industry in countries like Thailand. Therefore, uncovering tick-borne infections in the field, as well as assessing their clinical impact, is vital in formulating solutions to these diseases.

Herein, we report the detection of selected TBPs in dairy bovine samples from Thailand. The agents of tick fever, *B. bovis*, *B. bigemina,* and *A. marginale*, were identified. Thailand has long been considered an endemic country for tick fever (bovine babesiosis and anaplasmosis), with reported herd mortality rates of 0.5% and losses, mainly from mortalities, estimated at US$1.8 million in 1999 [27]. The predominant species of *Babesia* in cattle in Thailand are *B. bovis* and *B. bigemina*, the etiologic agents of bovine babesiosis. Both were observed at relatively lower rates (4.53% and 3.40%, respectively) than *A. marginale* (18.87%). The relatively high detection rates of *T. orientalis* in the current survey imply that this parasite is the predominant TBP among bovine herds in the sampled areas. The presently recorded *T. orientalis* overall detection rate (23.40%) was lower than previous surveys that reported 30.1–41.54% across Thailand [5,6,28]. The bovine infectious anemia outbreaks associated with the geographical spread of pathogenic *T. orientalis* have been documented in countries such as Australia, New Zealand, and recently, the USA [29,30,31,32]. As such, the widespread presence of *T. orientalis* in cattle, as demonstrated in this survey, signifies an enormous threat to the Thai dairy industry. Therefore, monitoring of *T. orientalis*, specifically the pathogenic Ikeda and Chitose types, should be conducted regularly, and infected animals should be provided proper treatment and management.

A majority of the positive samples were singly infected, while several samples were found to be positive for more than one TBP. A possible explanation for the single-TBP-positive samples is the competitive exclusion between various TBPs in cattle [33]. On the other hand, in endemic areas, coinfection of TBPs is a common occurrence, especially in cases where the pathogens are transmitted by the same vectors [34]. *B. bovis*, *B. bigemina*, and *A. marginale* have the same biological vector, the *Rhipicephalus* spp. tick, which is the predominant cattle tick in Thailand [35]. In this study, the coinfection combination of *T. orientalis* and *A. marginale* was the most frequently recorded. Remarkably, these two pathogens were the only noted combination not to exhibit competitive exclusion or negative interference within cattle [33]. A broader investigation is required to obtain absolute confirmation regarding this observation.

We demonstrated the detection of TBPs in dairy cattle from four provinces in northern and western (central) Thailand and recorded higher detection rates in samples from Lampang province. Bovine tick-borne infections have been extensively studied in northern and western (central) parts of Thailand in the past, where researchers reported the aforementioned pathogens in cattle [6,10,11,12,28,36,37], indicating their endemicity in the localities where they were detected. Studies on bovine TBDs have been performed in Lampang previously. *B. bovis* (8.33%) and *B. bigemina* (10.71%) detection in Lampang in this survey were lower than those reported by Cao et al. [10] and Simking et al. [11] but higher than those observed by Koonyosying et al. [28] and Yoshinari et al. [36]. Current *T. orientalis* (32.14%) and *A. marginale* (25%) detection were higher and lower than the detection rates of a previous Lampang survey of 21.05% and 85.53%, respectively [28]. On the other hand, we observed higher TBP detection rates than a report from Lamphun and lower than those from Nakhon Pathom [28]. The variances in the reports of TBPs may be attributed to different factors, such as the profiles of the animals (age, sex, breed, etc.) and external aspects, such as environmental factors (microclimate, tick abundance, and management practices of farmers) [38]. Worth noting in this study is the practice of free grazing in Lampang, where the highest rates were recorded. Grazing allows a longer exposure time to questing ticks in the pastures, increasing the risk of ectoparasite infestation and TBDs [6]. This may explain the higher rates in Lampang than in other provinces.

Anemia is one of the major indicators of TBDs. In this study, the detection of TBPs, specifically samples positive for BTH, *T. orientalis,* and *A. marginale,* was associated with significantly lower PCV values. This contradicts the results of previous Thai studies where the differences between the PCV values of cattle positive and negative for *T. orientalis* were trivial [5,28]. *T. orientalis*-infected cattle may show normal hematocrit values (24–46%) to as low as 8% [30,39], indicating that positive animals present varying hematologic indices. In cases of BTH- and *A. marginale*-infected cattle, the PCV values markedly decreased as well, similar to the findings of an investigation on cattle in India [40]. The significantly lower PCV values observed in infected cattle were within the lower normal ranges [26]. Although no overt disease was observed in the TBP-positive cattle in the present survey, there may be an impact on milk yield. Still, the impact of this finding on bovine productivity should not be ignored, as lower hematocrit caused by tick-borne infections can significantly reduce milk production in dairy animals [17,38,41]. This is a point to investigate in future studies.

We also assessed two diagnostic tools for the detection of TBPs: microscopic identification (MI) and PCR assays. Cohen’s kappa indices obtained in this study resulted in slight and fair agreement between the two tools, suggesting that microscopy may not be an adequate alternative to PCR in the detection of TBPs. Although traditionally used, MI of TBD agents is appropriate during the acute stage of the infections, when parasites are abundant in the bloodstream [42]. Although MI is the most convenient detection tool, it is not effective in detecting carrier or subclinical cattle due to the very low parasitemia [43,44,45]. Moreover, MI is highly reliant on the skill of the microscopist, leading to varied results and poor diagnosis [42]. On the other hand, the PCR test is said to be a thousand times more sensitive than microscopy; thus, it is currently the preferred pathogen identification tool for TBPs [43]. PCR tests and other nucleic acid-based tests in general can be adapted to a variety of conditions and highly specific targets, making them more reliable and accurate pathogen identification tools [46]. Laboratory reagents and equipment that are required to perform the tests can be costly for resource-constrained places, but the increasing accessibility to nucleic acid-based tests has contributed to lowering the cost of PCR tests. Therefore, PCR may soon make traditional detection tools, such as MI, obsolete.

There are several limitations to the present study. First, the study only focused on the northern and central/western parts of Thailand. It would have been more interesting if data from other parts, such as east and southern Thailand, were available, as this would have provided a broader insight into the impact of TBD on dairy animals across Thailand. Another limitation is the lack of molecular characterization of the detected TBPs, specifically for BTH-positive samples that were negative in the species-specific PCRs. In this study, the authors focused on reported piroplasma in Thailand, i.e., those that were screened using species-specific PCRs. Confirmation through sequencing is recommended in future studies. Finally, the status of the animals pertaining to other anemia-causing infections was not confirmed. Hence, this must be considered in interpreting the findings on PCV.

## 5. Conclusions

In this investigation, various TBPs, namely, *B. bovis*, *B. bigemina, T. orientalis,* and *A. marginale*, were detected in dairy cattle samples from the northern and western provinces of Thailand. Notably, *T. orientalis* and *A. marginale* were prevalent in the surveyed bovine herds. TBP-positive samples had significantly altered PCV values compared with those of the non-infected cattle. Moreover, the comparison between microscopy and PCR yielded a slight and fair agreement, indicating that results obtained by microscopy may not suitably replace TBP detection by PCR. Further studies on the impact of TBDs on the production and reproduction of Thai dairy cattle are warranted.

## Figures and Tables

**Table 1:** Background of dairy cattle farms surveyed in the current study.

Category	Province			
	Lampang	Lamphun	Nakhon Pathom	Kanchanaburi
	(N = 10)	(N = 8)	(N = 1)	(N = 1)
Herd size				
<20 head	2	2	0	0
20 to 50 head	6	5	0	0
>50 head	2	1	1	1
Farm management practices				
free grazing	10	0	n.d.a.	n.d.a.
stall feeding	0	8		
Presence of ticks in sampled animals				
yes	3	0	n.d.a.	n.d.a.
no	7	8		
Tick control methods				
yes	7	4	n.d.a.	n.d.a.
no	3	4		
TBD awareness				
yes	10	3	n.d.a.	n.d.a.
no	0	5		
Animal healthcare				
Livestock technician/veterinarian	0	0	n.d.a.	n.d.a.
farmer–owner	6	6		
both	4	2		

n.d.a.: no data available; N: number of sampled farms.

**Table 2:** Detection rates for tick-borne pathogens in cattle samples from dairy farms in northern and western Thailand.

Pathogen	Frequency (%)					
	Lampang	Lamphun	Nakhon Pathom	Kanchanaburi	Total	*p* Value
*Babesia/Theileria/Hepatozoon*	53/84 (63.10)	9/51 (17.65)	23/70 (32.86)	4/60 (6.67)	89/265 (33.58)	<0.001 ***
*B. bigemina*	9/84 (10.71)	2/51 (3.92)	1/70 (1.43)	n.d.	12/265 (4.53)	0.008 **
*B. bovis*	7/84 (8.33)	n.d.	1/70 (1.43)	1/60 (1.67)	9/265 (3.40)	0.024 *
*T. orientalis*	27/84 (32.14)	14/51 (27.45)	17/70 (24.29)	4/60 (6.67)	62/265 (23.40)	0.004 **
*A. marginale*	21/84 (25.00)	1/51 (1.96)	17/70 (24.29)	11/60 (18.33)	50/265 (18.87)	0.005 **

n.d.: not detected; * *p* ≤ 0.05; ** *p* ≤ 0.01; *** *p* ≤ 0.001.

**Table 3:** Infection types of positive samples based on species-specific PCR results.

Single Infections		Dual Infections		Triple Infections		Quadruple Infections	
*B. bigemina* (Bbi)	4	Bbi and Bbo	1	Bbi, Bbo, and Ama	1	Bbi, Bbo, Tor, and Ama	1
*B. bovis* (Bbo)	1	Bbi and Tor	1	Bbi, Tor, and Ama	2		
*T. orientalis* (Tor)	42	Bbo and Tor	1	Bbo, Tor, and Ama	2		
*A. marginale* (Ama)	27	Bbi and Ama	2				
		Bbo and Ama	2				
		Tor and Ama	13				

**Table 4:** Agreement analysis between PCR and microscopic examination results.

Microscopy	PCR							
	BTH 18S rRNA		*B. bigemina* and *B. bovis*		*T. orientalis*		*A. marginale*	
	Positive	Negative	Positive	Negative	Positive	Negative	Positive	Negative
Positive	19	17	2	11	14	20	20	30
Negative	40	100	9	154	27	115	25	101
Cohen’s kappa (κ)	0.20		0.11		0.21		0.21	
SE	0.075		0.108		0.084		0.079	
95% CI	0.048–0.343		−0.105–0.317		0.042–0.369		0.052–0.363	

SE: standard error; CI: confidence interval; κ interpretation: none (<0), slight (0–0.20), fair (0.21–0.40), moderate (0.41–0.60), substantial (0.61–0.80), and almost perfect (0.81–1.00) [25].

**Table 5:** Changes in the packed cell volume of PCR-positive and PCR-negative cattle samples in this study.

Pathogen	Positive			Negative			*p* Value
	N	Mean PCV ± SD (%)	95% CI	N	Mean PCV ± SD (%)	95% CI	
At least 1 TBP	126	27.59 ± 5.21	26.67–28.51	138	30.54 ± 4.52	29.78–31.30	<0.001 ***
*Babesia/Theileria/Hepatozoon*	88	27.18 ± 5.39	26.03–28.32	176	30.11 ± 4.62	29.43−30.80	<0.001 ***
*T. orientalis*	62	26.90 ± 5.44	25.52–28.28	202	29.82 ± 4.76	29.16−30.48	<0.001 ***
*A. marginale*	49	27.84 ± 4.92	26.43–29.26	215	29.43 ± 5.07	28.75−30.11	0.049 *

N: number of samples; PCV: packed cell volume; SD: standard deviation; CI: confidence interval; * *p* ≤ 0.05; *** *p* ≤ 0.001; normal range for bovine PCV: 24.0–46.0% [26].

## Data Availability

The datasets generated during and/or analyzed during the current study are available from the corresponding author upon reasonable request.

## Supplementary Materials

The following supporting material can be downloaded at https://www.mdpi.com/article/10.3390/ani13182844/s1. Table S1. PCR assays used in the current study.
